# Case report: Successful treatment of renal-limited thrombotic microangiopathy secondary to chronic lymphocytic leukemia

**DOI:** 10.3389/fneph.2024.1400027

**Published:** 2024-08-13

**Authors:** Kristina Nasr, Sabine Karam, Marshall Mazepa, Jan Czyzyk, Nattawat Klomjit

**Affiliations:** ^1^ Division of Nephrology and Hypertension, Department of Medicine, University of Minnesota, Minneapolis, MN, United States; ^2^ Division of Hematology, Oncology and Transplantation, Department of Medicine, University of Minnesota, Minneapolis, MN, United States; ^3^ Department of Laboratory Medicine and Pathology, University of Minnesota, Minneapolis, MN, United States

**Keywords:** chronic lymphocytic leukemia, CLL, thrombotic microangiopathy, TMA, nephrotic syndrome

## Abstract

Thrombotic microangiopathy (TMA) is a rare renal complication of patients with chronic lymphocytic leukemia (CLL) and is often associated with peripheral features. We present the first case of CLL patients with renal-limited TMA. A 70-year-old female patient with a history of well-controlled type 2 diabetes and baseline albuminuria of 87.2 mg/g 1 year prior and CLL was on active surveillance only. Her baseline white blood cell (WBC) was 202.6 x 10^3^/µl. She presented with nephrotic syndrome with proteinuria of 10 g/g and a subsequent unremarkable serologic work-up. A kidney biopsy revealed diabetic glomerulosclerosis and chronic TMA. Initially, she was treated conservatively with angiotensin receptor blockade and sodium glucose cotransporter-2 inhibition but progressed with increased proteinuria of 17 g/g. Complement functional panel testing was pursued and showed dysregulation of the classical and alternative complement pathways. We decided to treat CLL which was suspected to be the culprit. At 9 months post-ibrutinib initiation, there was a 90% reduction in the WBC as well as a 94% reduction in proteinuria (17 g/g to 0.97 g/g). This case emphasizes the role of complement dysregulation in the pathogenesis of TMA in CLL patients. Treatment of CLL can restore complement dysregulation and improve renal outcomes.

## Introduction

Chronic lymphocytic leukemia (CLL) is an indolent malignancy caused by the over-production of mature but dysfunctional B lymphocytes and comprises 25% to 30% of total leukemias in the United States. CLL is mostly prevalent in the adult population with an average age at diagnosis of 70 years (1). CLL primarily manifests in the peripheral blood, spleen, lymph nodes, and bone marrow (1). It rarely involves extra-medullary or extra-nodal organs with 10% of the cases affecting the genitourinary system, including the kidneys (2). Thrombotic microangiopathy (TMA) is a rare condition where endothelial injury leads to systemic microthrombi (3). Most patients have peripheral features including microangiopathic hemolytic anemia and thrombocytopenia. The kidney is one of the most affected organs in TMA; and certain patients may only present with renal-limited TMA in the absence of peripheral features (4). TMA rarely occurs in CLL and the mechanism remains unclear. Thus far, there have been fewer than 10 case reports of CLL patients with TMA lesions on a kidney biopsy (2, 5, 6). Notably, all cases harbored peripheral features. We present a case of CLL who was under active surveillance but developed renal-limited TMA secondary to dysregulated classical and alternative complement pathways.

## Case description

**Figure 1:**
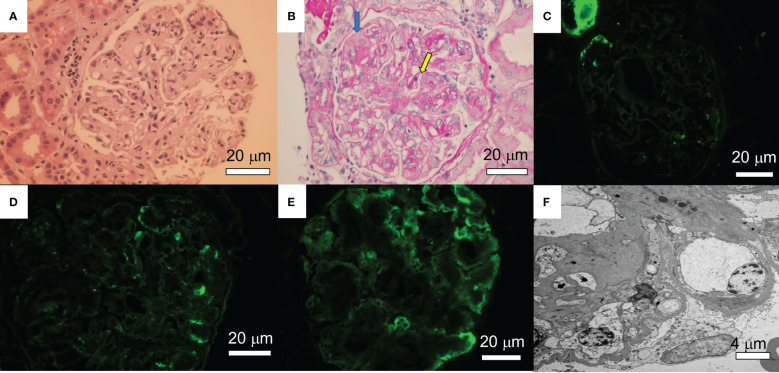
Renal pathology findings. **(A)** A glomerulus shows mesangial matrix expansion and hypercellularity. A few loops contain neutrophils. Crescents and fibrinoid necrosis are not present (Hematoxylin&Eosin). **(B)** A glomerulus shows PAS-positive mesangial nodules with lobular accentuation and focal double contouring (yellow arrow). There is some focal endothelial swelling (blue arrow) (Periodic Acid-Shiff: PAS). **(C-E)** Immunofluorescence studies following pronase digestion, the glomeruli are negative for lambda **(C)**, IgG **(D)**, and C3 **(E)**. **(F)** Electron microscopy showed subendothelial expansion with cellular elements and fluffy material. Electron dense deposit is absent. Segments of double contours are present. A few loops show infiltrating leukocytes. The glomerular basement membranes appear thickened.

**Figure 2:**
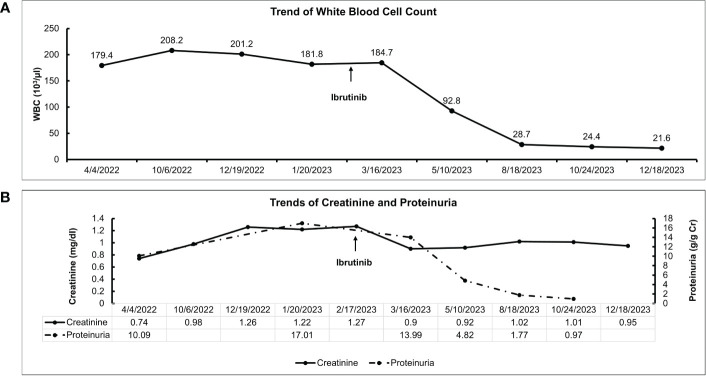
Clinical course and treatment response of the patient. **(A)** Trend of white blood cell count after ibrutinib initiation. **(B)** Trends of serum creatinine and proteinuria after ibrutinib initiation.

A 70-year-old female patient has a history of type 2 diabetes (well-controlled over the past 10 years and no signs of neuropathy or retinopathy), hyperlipidemia, hypertension (well-controlled with 25 mg losartan daily), and CLL stage 2 diagnosed approximately 8 years prior and had been under active surveillance until her presentation to our nephrology clinic. She was referred due to a concern for nephrotic syndrome. She had a history of mild albuminuria with a urine albumin creatinine ratio (UACR) of 87.2 mg/g measured 1 year prior to the referral. Upon presentation, her blood pressure was 135/81 mmHg and she had 1+ bilateral lower extremity pitting edema. She also had evidence of hepatosplenomegaly and lymphadenopathy. Laboratory studies showed a serum creatinine (SCr) of 0.84 mg/dl, serum albumin (SAlb) of 3.4 mg/dl, hemoglobin (Hb) of 11.8 g/dl, a white blood cell count (WBC) of 202.6 x 10^3^/µl, an absolute lymphocyte count of 186.4x10^3^/µl, a platelet count of 186 x 10^3^/µl, and an HbA1c of 7.2 mmol/l. Urinalysis showed 23 red blood cells per high power field (hpf) and 13 WBC/hpf. The urine protein creatinine ratio (UPCR) was 10.09 g/g. A serological work-up that included antineutrophil antibody (ANA), C3, C4, anti-neutrophilic cytoplasmic antibody (ANCA), and phospholipase A2 receptor antibody was negative. Screening for monoclonal proteins and cryoglobulins was negative. Hepatitis B and C and HIV serologies were negative. Detailed laboratory tests results are shown in **Table 1**. She subsequently underwent a kidney biopsy which showed mesangial expansion with an increase in matrix and cellularity resulting in PAS-positive mesangial nodules. Additionally, the patient had thickened capillary walls and micro aneurysms. These findings are typical for early diabetic nephropathy (7). However, double contouring of the glomerular basement membrane (GBM) was also noted, which was atypical for diabetic nephropathy (7). Immunofluorescence was negative for any light chain restriction process, C3, and C1q. C4d staining was not performed. Electron microscopy showed segmental foot process effacement thus ruling out the possibility of “primary podocytopathy” such as minimal change disease (MCD) or primary focal segmental glomerulosclerosis (FSGS). However, segmental foot process alone cannot differentiate diabetic nephropathy from TMA. Several capillary loops showed subendothelial expansion with cellular elements and fluffy material. These features, along with the double contouring of the GBM, were strongly suggestive of endothelial injury and TMA, and are atypical in diabetic nephropathy. There were no electron-dense deposits or fibrils (**Figure 1**). Hence, these findings were consistent with both diabetic glomerulosclerosis and TMA. Further laboratory investigations showed normal lactate dehydrogenase (LDH) of 162 U/l, normal haptoglobin, bilirubin 0.4 mg/dl, and platelet 184 x10^3^/µl (prior values were always >150 x10^3^/µl) with the absence of schistocytes on peripheral blood smear. Urinalysis showed the presence of microscopic hematuria. ADAMTS13 function and an antiphospholipid syndrome panel were normal. However, the complement functional panel showed dysregulated activity of C3 and C5 convertase and depleted classical and alternative pathways (**Table 2**). Further complement studies showed that the patient was negative for C3, C4, and C5 nephritic factors. Genetic testing for complement gene variants was negative. Initially, the patient was started on diuretics, a maximally tolerated dose of losartan, and an SGLT2 inhibitor. Despite 3 months of optimized conservative therapy, she deteriorated with worsening edema and uncontrolled hypertension. Her SCr increased to 1.27 mg/dl. The SAlb decreased to 3.0 mg/dl and the UPCR escalated to 17.0 g/g. Given the negative work-up for other causes of TMA, we suspected that CLL was the culprit for the renal-limited TMA. Prior to the treatment, flow cytometry was done and showed a CD5+ B-cell population with probable kappa light chain restriction. The malignant cells also expressed CD19, CD20, and CD23. Cytogenetics showed no evidence of loss of MYB, ATM, D13S319, or TP53. There was no gain of chromosome 12 nor IGH/CCND1 fusion. After a discussion with the patient’s hematologist, we decided to start ibrutinib for CLL based on the side effects profile and the patient’s preference. After therapy commencement, her blood pressure became controlled with a complete resolution of the edema. Her WBC count progressively decreased (**Figure 2A**) along with an overall improvement of her SCr and urine protein levels (**Figure 2B**). At 9 months post-treatment, labs showed a SCr of 0.95 mg/dl, a SAlb of 4.3 mg/dl, and a UPCR of 0.97 g/g. Complement functional panel post-treatment showed no evidence of complement activation or dysregulation (**Table 2**). The patient reported tolerating the treatment well without any side effects.

**Table 1:** Baseline laboratory characteristics.

Laboratories	Results
Serum creatinine (mg/dl)	0.84
White blood cell count (cellsx10^3^/µl)	202.6
Hemoglobin (g/dl)	11.8
Platelets (cellsx10^3^/µl)	186
Serological work-up	
ANA	Negative
Complement C3 (mg/dl)	111
Complement C4 (mg/dl)	26
PR3-ANCA	Negative
MPO-ANCA	Negative
PLA2RAb	Negative
Viral panel (Hepatitis B surface antigen, Hepatitis C virus antibody, and Human immunodeficiency virus antibody)	Negative
Monoclonal protein testing	
Serum protein electrophoresis	Negative
Serum immunofixation	Negative
Kappa FLC (mg/dl)	2.39
Lambda FLC (mg/dl)	7.10
Kappa/Lambda Ratio	0.34

ANA, antinuclear antibody; PR3, proteinase 3; MPO, myeloperoxidase; ANCA, anti-neutrophilic cytoplasmic antibody; SPEP, serum protein electrophoresis; PLA2RAb, phospholipase A2 receptor antibody.

**Table 2:** Functional complement assay pre- and post-ibrutinib therapy.

Complement proteins and functions	Pre-treatment	Post-treatment
CH50 (41-95 Units/mL)	<12	97
Alternative pathway function assay (50-130%)	13%	76%
Fluid phase activity assay (<7.5%)	11.1%	4.6%
FH autoantibody (<200AU)	<50	57
FB autoantibody (<200AU)	<50	86
Complement C3 level (90-180 mg/dl)	83	137
Complement C4 level (15-47 mg/dl)	18	46
FB level (22-50 mg/dl)	41.1	37.0
Ba level (<1.2 mg/l)	2.7	1.1
Bb fragment level (<2.2 mg/l)	2.3	0.9
Factor D level (0.78-1.59 mg/l)	1.99	1.14
Properdin level (10-33 mg/l)	4.3	16.6
Complement C5 level (13.5-7 mg/dl)	18.1	20.4
sC5b-9 (<0.3 mg/l)	0.62	0.13
ADAMTS-13 activity (>60%)	>104%	NA

CH50, 50% hemolytic complement; FH, factor H; FB, factor B; sC5b-9, soluble membrane attack complex; ADAMTS-13, A disintegrin and metalloproteinase with thrombospondin-1 motifs.

## Discussion

Our case demonstrated that renal-limited TMA is one of the rare renal manifestations of CLL. Although the patient has some pathological findings of early diabetic nephropathy, she also harbors certain indications of TMA, which include subendothelial swelling containing cellular and fluffy material, loss of endothelial fenestration, and double contouring of the GBM (8). Renal-limited TMA likely contributes to the nephrotic syndrome in this case more than diabetic nephropathy due to the well-controlled nature of her diabetes and only early diabetic changes in the glomeruli. Segmental foot process effacement, mild hypoalbuminemia, and gradual onset of nephrotic syndrome favor the secondary podocytopathy process from TMA than primary podocytopathy as seen in MCD or primary FSGS (8). In this case, we also showed that activation of the alternative and classical complement pathways were the culprit, and that treating CLL can result in dramatic improvement of renal outcomes. In one of the largest studies describing the renal pathology of CLL patients, approximately 7.5% of patients have kidney disease upon diagnosis and 16.2% develop kidney disease during their clinical course (2). However, only 1.2% of the patients require a kidney biopsy. Patients typically presented with renal insufficiency or nephrotic syndrome (2). Kidney diseases in patients with CLL may occur through diverse mechanisms including extrarenal obstruction, intra-parenchymal leukemic infiltration, tumor lysis syndrome, glomerular diseases, and medication side effects (9). Therefore it is important to identify the cause of kidney disease in CLL patients since kidney disease can reduce survival independently of CLL burden (9). Many glomerular and tubulointerstitial lesions in CLL are frequently associated with the presence of monoclonal gammopathy such as immunotactoid glomerulopathy, cryoglobulinemic glomerulonephritis (GN), TMA, C3 Glomerulonephritis (C3GN), and light chain proximal tubulopathy (2, 9, 10). However, several non-monoclonal protein related kidney diseases have also been reported, namely membranoproliferative glomerulonephritis (MPGN), minimal change disease, FSGS, membranous nephropathy, AA amyloidosis, and fibrillary GN (9).

TMA characterizes a diverse group of diseases depicted by primary microvascular endothelial cell injury. Microvascular injury leads to platelet activation and eventually, the formation of platelet-rich and/or fibrin thrombi that occlude small vessels of various organs, primarily the kidneys and nervous system (3). Approximately 50-75% of patients with biopsy-confirmed renal TMA have peripheral features (8).The etiology of TMA is diverse and often categorized as primary versus secondary. Primary TMA is rare and often caused by complement disorders (3). On the other hand, the more common secondary TMA can be caused by drugs, neoplasms, monoclonal protein, infections, malignant hypertension, autoimmune diseases, pregnancy, and transplantation (stem cell or solid organ) (11–13). Also, in those with genetic susceptibility, secondary causes may trigger primary TMA (14).

In CLL, most cases with TMA harbor monoclonal proteins which are thought to incite TMA (5, 6). These toxic monoclonal proteins may lead to endothelial injury resulting in increased susceptibility to microthrombi formation and eventually TMA (13). Another plausible mechanism involves dysregulation of the alternative complement pathway where immunoglobulins act as an autoantibody to a complement-regulating protein, such as complement factor H or factor B (4, 13). Furthermore, concomitant use of thrombogenic chemotherapy such as pentostatin or a post-stem cell transplant state may induce TMA in certain CLL cases (2). As opposed to previous case reports where all patients had peripheral features, our patient had renal-limited TMA only. Moreover, a monoclonal protein was not identified in our case nor were secondary causes such as drugs or autoimmune disorders. Genetic testing was also negative for complement disorders. However, our patient had striking evidence of classical and alternative complement pathway activation as shown by suppressed C3, C4, CH50, and elevated soluble membrane attack complex (sMAC) levels. Patients with CLL have been shown to have complement dysregulation which may explain the susceptibility to infection in those patients (15). Recently, it has been shown that CLL patients have consistent low-grade classical pathway activation via the formation of IgG-hexamers (IgG aggregates) (16). Despite this association, TMA in CLL patients remains a rare problem and is not often present unless CLL is advanced. Moreover, though our patient had a CLL diagnosis, she did not meet the criteria for treatment from a hematological standpoint. This was strikingly different from most previous case reports where patients met the criteria for treatment based on hematological burden. As we suspected that her kidney disease was a direct consequence of her CLL, we decided to proceed with CLL treatment. The phenomenon in this case somewhat resembles monoclonal gammopathy of renal significance (MGRS), where a toxic monoclonal protein damages the kidneys with a low disease burden (17). One could wonder whether this case could be a case of CLL with renal significance.

In neoplasm-associated TMA, treatment should focus on the treatment of the underlying neoplasm to address the primary driver of TMA. Therapeutic plasma exchange or eculizumab (C5 inhibitor) has been used with mixed results (18). In our case, the patient was treated with ibrutinib, a bruton kinase inhibitor, that inhibits cell growth, proliferation, and survival of B-cells (19). In the absence of any known mutations, the choice of treatment with ibrutinib was based on the side effects profile and after shared decision-making with the patient. At 9 months post-treatment, the patient had a dramatic response with a 90% reduction in WBCs as well as a 94% reduction in proteinuria (17 g/g to 0.97 g/g). The reduction in lymphocytes was associated with an improvement in proteinuria and complement function as shown in **Figure 2**. This improvement in proteinuria supports the link between renal-limited TMA and CLL in this case. Although ibrutinib did not directly affect the complement system, it controlled CLL and decreased the pathological lymphocytes. Therefore, treating CLL would lead to an improvement in the complement dysregulation and alleviate kidney injury.

In conclusion, we report the first case of renal-limited TMA in a CLL patient who was under active surveillance. Our case demonstrates the pivotal role of complement dysregulation via classical and alternative pathways in CLL-related TMA. CLL treatment can lead to the complete resolution of complement dysregulation and a dramatic improvement in kidney outcomes. Clinicians need to be aware of this potential complication of CLL to promptly diagnose and implement appropriate therapy. Future studies are needed to identify risk factors and long-term clinical outcomes in TMA among CLL patients.

## Data Availability

The original contributions presented in the study are included in the article/supplementary material. Further inquiries can be directed to the corresponding author.
